# Exploring Relations Between Psychological Capital, Social Support and Self‐Compassion Among Siblings of Individuals With Developmental Disabilities

**DOI:** 10.1111/cch.70356

**Published:** 2026-09-27

**Authors:** Batel Hazan‐Liran, Ofra Walter

**Affiliations:** ^1^ Faculty of Education Tel‐Hai University of Kiryat Shmona in the Galilee (THU) Qiryat Shemona Israel

**Keywords:** family dynamics, mediation analysis, psychological capital, quantitative research, self‐compassion, siblings, social support, special needs

## Abstract

**Background:**

We examined correlations between psychological capital (PsyCap), social support and self‐compassion among siblings of individuals with developmental disabilities versus siblings of individuals without developmental disabilities.

**Methods:**

We hypothesized that (1) there would be positive correlations between PsyCap, social support and self‐compassion at the sample level and within each group; (2) siblings of individuals without developmental disabilities would exhibit lower levels of these variables than siblings of individuals with developmental disabilities; and (3) relations between social support and self‐compassion would be mediated by PsyCap in both groups. We surveyed 154 adults aged 18–71 years, including 42 siblings of individuals with developmental disabilities (19–71 years) and 112 siblings of individuals without developmental disabilities (18–71 years).

**Results:**

Results indicated significant positive correlations between PsyCap, social support and self‐compassion at the sample level and among siblings of individuals without developmental disabilities. As hypothesized, PsyCap mediated relations between social support and self‐compassion. Contrary to expectations, no significant differences were found between the two groups in levels of PsyCap, social support or self‐compassion.

**Conclusion:**

These findings suggest that PsyCap constitutes a key internal psychological resource that supports self‐compassion among siblings, including those of individuals with developmental disabilities, highlighting its potential value as a target for interventions promoting sibling well‐being.

## Summary

- Psychological capital (PsyCap) mediates relations between social support and self‐compassion in siblings of individuals with developmental disabilities.
- Siblings of individuals with developmental disabilities and siblings of individuals without developmental disabilities exhibit similar levels of PsyCap, social support and self‐compassion.
- Findings suggest that tailored interventions focusing on PsyCap could enhance self‐compassion and well‐being.

## 1 Introduction

Sibling relationships are among the longest‐lasting relationships within the family and play a central role in social and emotional development, often serving as sources of companionship, emotional support and social comparison (McHale et al. 2006; Tomeny et al. 2016). When a family includes a child with developmental disabilities, family routines, roles and resources frequently require substantial adjustment (Hartling et al. 2010). These changes may increase psychological demands on siblings, placing them at greater risk for emotional difficulties, including anxiety, depression and psychological distress (Tomeny et al. 2016; Vermaes et al. 2011; Walton and Ingersoll 2015).

Social support from family, friends and community resources is a well‐established protective factor helping individuals cope with stress. It enhances social and emotional well‐being, strengthens self‐esteem and promotes psychological resources such as emotional intelligence and psychological capital (PsyCap) (Fabio and Kenny 2011; Garrido et al. 2020; Hartling et al. 2010; Luthans et al. 2013; Meltzer 2021; Siedlecki et al. 2013; Tomlinson et al. 2016). Within families of individuals with developmental disabilities, social support has also been associated with better family adaptation and fewer emotional and behavioural difficulties among siblings (Ekas et al. 2010).

Another resource that may help individuals cope with stress is self‐compassion, encompassing self‐kindness, common humanity and mindfulness. Self‐compassion has been consistently associated with psychological well‐being by facilitating adaptive emotion regulation and resilience (Neff 2003a; Neff and Davidson 2016). Previous studies in various populations have also demonstrated positive associations between self‐compassion, PsyCap and social support (Lowery and Cassidy 2022; Poots and Cassidy 2020). However, to the best of our knowledge, the role of PsyCap has not been examined among siblings of individuals with developmental disabilities.

Existing research on siblings has focused on adjustment, well‐being and other psychological outcomes, leaving the potential contribution of PsyCap as a psychological resource supporting siblings' adaptation and well‐being largely unexplored. The present study was designed to address this gap by comparatively examining PsyCap, perceived social support and self‐compassion among siblings of individuals with and without developmental disabilities. Specifically, we explored the associations among these variables and examined whether PsyCap mediated relations between social support and self‐compassion. We hypothesized positive associations among PsyCap, social support and self‐compassion, with higher levels of these resources among siblings of individuals with developmental disabilities. The goal was to contribute to a deeper understanding of the psychological resources that promote siblings' well‐being.

## 2 Literature Review

### 2.1 Sibling of an Individual With Developmental Disabilities

Sibling relationships last throughout the life course (Tomeny et al. 2016) and are shaped by numerous factors, from child characteristics to cultural norms and values (McHale et al. 2006). Living in a family with an individual with developmental disabilities can create numerous stressors for siblings without disabilities. They receive less parental attention and feel a duty to care for their siblings. They take on parental roles, putting them at risk for anxiety, depression and anger (Tomeny et al. 2016; Vermaes et al. 2011; Walton and Ingersoll 2015). Living with an individual with developmental disabilities can also have positive effects. Siblings of these individuals are more responsible, empathetic, mature and prosocial and possess a stronger sense of self‐efficacy than siblings of individuals without developmental disabilities (Tomeny et al. 2016; Vermaes et al. 2011; Walton and Ingersoll 2015).

### 2.2 Social Support of Siblings of Individuals With Developmental Disabilities

Social support is defined as the experience that leads people to believe they are cared for, loved, valued and part of a network of mutual obligations (Cobb 1976). Sources of support can include partners, family members, friends, coworkers, social and community connections—even a beloved pet (Meltzer 2021). Support can manifest in various ways, including offering advice, providing financial assistance, helping with tasks, assisting in caregiving and providing warmth. Sometimes, it is enough to have the sense that these resources are available, and there is someone to turn to (Taylor 2011).

A study exploring psychological adaptation among international students found positive correlations between social support and psychological adaptation, with the source of support not significantly important (Bender et al. 2019). In another study, social support was positively associated with higher levels of emotional intelligence among high school students (Fabio and Kenny 2011). Formal support programmes provided by health and welfare organizations and informal support from family and friends can significantly improve the mental well‐being of families of individuals with developmental disabilities (Ekas et al. 2010; Roberts et al. 2015). Studies conducted among siblings of individuals with developmental disabilities suggest social support helps them cope with stressors and reduces emotional and behavioural difficulties (Hartling et al. 2010; Roberts et al. 2015).

In addition to support received from parents, extended family, friends and peers, siblings of individuals with developmental disabilities may benefit from structured support provided by schools, healthcare professionals and community organizations. Sibling‐specific interventions, including psychoeducational programmes, peer support groups and family‐centred services, have been shown to enhance coping skills, reduce feelings of isolation and promote psychological well‐being (Hartling et al. 2010; Meltzer 2021; Roberts et al. 2015). These programmes provide opportunities for siblings to share experiences with peers facing similar family circumstances, normalize their emotional responses, develop adaptive coping strategies and strengthen resilience. Despite the growing recognition of these interventions, relatively little research has examined the psychological mechanisms through which such support contributes to siblings' well‐being, highlighting the importance of investigating protective resources such as PsyCap.

### 2.3 PsyCap and Siblings of Individuals With Developmental Disabilities

PsyCap is an individual's positive and developmental state of mind, comprising four components (Luthans 2002a, 2002b; Luthans and Youssef‐Morgan 2017). *Self‐efficacy* is expressed in self‐confidence and a belief in the ability to complete challenging tasks, formed through experiences where individuals feel in control of the outcomes (Bandura 1997). *Optimism* is expressed in positive attributions regarding current and future successes (Luthans et al. 2007). *Hope* is expressed in willpower, initiative and the ability to find alternative pathways, even when other factors delay the achievement of goals (Snyder et al. 1991). *Resilience* helps individuals cope with difficulties; it is not innate but part of the personality that develops with experiences of both positive and negative events (Luthans 2002a, 2002b).

Little research has specifically examined PsyCap among siblings of individuals with developmental disabilities, but some work has touched on it, looking at other family members and the components of PsyCap. A study examining the experiences of mothers of individuals with developmental disabilities highlighted the importance of optimism to bolster family strengths (Joshi 2021). Another study found parents shaped their perceptions of the parental role with the help of optimistic attitudes, and these provided hope (King et al. 2009). A study examining the protective and risk factors for siblings of individuals with autism found they had a higher sense of self‐efficacy than the control group (Walton and Ingersoll 2015). A study of families of children with developmental disabilities explored the perspective of healthy adolescent siblings and found that the level of family resilience was significantly influenced by the sibling relationship; the more positive the relationship, the higher the family resilience (Retno et al. 2019). A survey conducted among parents and primary caregivers of children with autism found resilience characteristics in these families, manifested in the attribution of positive meaning to the child's disability, the mobilization of necessary resources, family closeness, significant appreciation for life and spiritual strength (Bayat 2007).

### 2.4 PsyCap and Social Support

A study among students found PsyCap was a mediating factor between social support and subjective well‐being (Li et al. 2014). Another study with a student sample considered relations between PsyCap, social support and career capital (i.e., talent, knowledge and skills based on education and personal experiences; see Pryce‐Jones 2010) and found strong positive relations between PsyCap and social support, whereas social support was a mediator of PsyCap and career capital (Jo Ching et al. 2019). A study among women found strong and positive relations between social support and PsyCap, whereas social support and PsyCap both predicted psychological well‐being (Mishra and Shafiq 2018).

The various components of PsyCap have been linked to social support in the context of families of children with developmental disabilities. A study on relations between social support and optimism among mothers of children with autism found that the more family support the mothers received, the higher their level of optimism (Ekas et al. 2010). Other researchers found the availability of social support resources significantly contributed to hope and resilience among parents of children with special needs (Dey and Amponsah 2020; Kausar et al. 2003). A study on parents of children with intellectual disabilities found that social support influenced parents' level of resilience (Mohan and Kulkarni 2018). Another study reported positive associations between perceived social support and self‐efficacy among mental health professionals (Wang et al. 2015).

Based on the literature, we argued that siblings of individuals with developmental disabilities who have higher levels of PsyCap and social support will be better able to deal with the challenges of their unique situation than those with lower levels.

### 2.5 Self‐Compassion and Siblings of Individuals With Developmental Disabilities

Self‐compassion consists of three main components (Neff 2003a). *Self‐kindness* is the ability to be kind to oneself in times of pain or failure, avoiding self‐criticism. *Common humanity* refers to perceiving one's experiences as part of the collective human experience, not as separate and individual. *Mindfulness* refers to holding painful thoughts and feelings in balanced awareness, rather than overidentifying with them. A combination of these components results in a positive, emotion‐based self‐approach that protects individuals from the negative consequences of self‐judgement, social isolation, painful thoughts and tendencies towards narcissism, self‐centredness and unhelpful social comparisons.

Self‐compassion helps individuals respond to distress in a balanced and mindful way, without avoiding or ignoring unpleasant feelings, even when their suffering stems from mistakes, failures or personal limitations. Researchers have found connections between self‐compassion and well‐being, self‐esteem, motivation, health, body image and interpersonal relationships (Neff and Davidson 2016). Barnard and Curry (2011) reviewed studies in the fields of personality, social psychology and clinical psychology that were linked to self‐compassion. The found self‐compassion had positive relations with satisfaction, well‐being, emotional intelligence and motivation and negative relations with anxiety, depression, avoidance, thought suppression, rumination, procrastination and perfectionism.

A study conducted among adults whose parents had mental disorders found that self‐compassion was a useful and important resource, but some found it difficult to develop because they had emotional barriers in their social and family lives (Dunkley‐Smith et al. 2021). There was a tension between the cognitive perception of self‐compassion and its actual implementation. Studies on parents of children with autism have found positive relations between self‐compassion and well‐being, hope, life satisfaction, goal commitment and quality of life; the higher the self‐compassion, the higher the levels of all variables (Bohadana et al. 2019; Neff and Faso 2015; Torbet et al. 2019). Researchers have also reported negative relations between self‐compassion and stress, depression and distress, leading to the conclusion that self‐compassion is a significant protective factor that can help parents of children with developmental disabilities cope with challenges. We argued that this protection should extend to the siblings of individuals with developmental disabilities.

Some research suggests having a sibling with a disability may lead to elevated levels of empathy and compassion (Borelli 2023). Persons in this situation often develop heightened sensitivity to the needs and emotions of others, fostering stronger interpersonal skills and emotional intelligence. This enhanced empathy and compassion can be a direct result of the increased caregiving roles assumed by siblings, as these roles necessitate a deeper understanding of and responsiveness to the needs of others.

At the same time, the experience has challenges. When a family member has an addiction, illness or disability, some adolescents are forced to care for and ensure the general functioning of this family member, putting their own needs aside. Studies conducted among this population found low levels of self‐compassion, despite the importance and value they attributed to the caring role (Berardini et al. 2021, 2022). Negative psychosocial outcomes can include internalized emotions, such as anger, fear and depression, as well as externalized behaviours like academic difficulties and conflicts with parents and siblings (Peterson 2017). The emotional toll of witnessing a sibling struggle and the additional attention siblings with a developmental disability receive from parents can lead to feelings of neglect or resentment, manifested in behavioural issues or a decline in academic performance (Amalia and Rositawati 2020).

Self‐compassion may play a crucial role by enhancing individuals' future orientation. The cultivation of self‐compassion helps to mitigate negative outcomes and fosters a healthier psychosocial environment. When siblings of individuals with developmental disabilities have self‐compassion, they are better equipped to handle the challenges of their unique family dynamics. By fostering an attitude of self‐kindness and understanding, they can navigate their own emotional experiences, reducing feelings of anger and depression. Not least, self‐compassion promotes resilience and adaptive coping strategies. By understanding and integrating these dynamics, we can better support the well‐being of siblings of individuals with developmental disabilities.

### 2.6 Self‐Compassion, PsyCap and Social Support

A study of parents of children with developmental disabilities found indirect relations between self‐compassion and resilience through the mediation of hope (Sünbül and Fakültesi 2020), with significant positive relations between self‐compassion and hope and also between resilience and hope. The researchers concluded self‐compassion significantly contributes to the levels of hope among parents of children with developmental disabilities. An intervention study aimed at helping individuals at risk for depression practice self‐compassion and optimism assessed participants' levels of happiness and depression at the intervention (Shapira and Mongrain 2010) and over time. There was a significant increase in happiness after 6 months and a notable decrease in depression lasting up to 3 months. A study comparing students and nonstudents found significant positive relations between self‐compassion and self‐efficacy (Liao et al. 2021), and a study among high school students found that personal skills and self‐efficacy significantly impacted self‐compassion (Wibowo et al. 2022). Self‐compassion stems from building a positive view of oneself and can be helpful in difficult times (Bernard et al. 2011; Neff 2003a).

Social support has been found to predict positive self‐esteem, thereby linking it to self‐compassion (Tomlinson et al. 2016; Wilson et al. 2020). A study among students examined relations between academic stress and well‐being through the mediation of self‐compassion, PsyCap and social support (Poots and Cassidy 2020). Researchers found negative relations between academic stress and well‐being and highlighted the importance of the mediation of self‐compassion, PsyCap and social support. Additionally, PsyCap predicted self‐compassion, and social support predicted higher PsyCap. A study among firefighters, police officers and emergency medical personnel considered the roles of PsyCap, self‐compassion, social support, relationship satisfaction and physical activity in relation to health and well‐being (Lowery and Cassidy 2022). All variables served as key mediating factors affecting health and well‐being.

Although PsyCap has been widely associated with psychological well‐being, resilience and adaptive coping across diverse populations, including students, employees, caregivers and individuals facing chronic stress (Luthans et al. 2007; Luthans et al. 2015), to the best of our knowledge, no studies have specifically examined PsyCap among siblings of individuals with developmental disabilities. This represents an important gap in the literature, as siblings often have unique lifelong emotional, social and caregiving challenges. Examining PsyCap in this population may therefore contribute to a better understanding of the psychological resources that promote adaptive functioning and well‐being.

### 2.7 The Present Study

We argued that the relations between the various components of PsyCap, social support and self‐compassion could benefit the siblings of individuals with developmental disabilities. We examined two groups: siblings of individuals with developmental disabilities and siblings of individuals without developmental disabilities.

Hypothesis 1 *PsyCap, social support and self‐compassion will be positively correlated both at the sample level and in each group (siblings of individuals with and without developmental disabilities) separately*.

Hypothesis 2 *There will be differences between groups in PsyCap, social support and self‐compassion, whereby siblings of individuals without developmental disabilities will have lower levels of all three variables*.

Hypothesis 3 *The relations between social support and self‐compassion will be mediated by PsyCap in both groups, whereby greater social support will lead to higher PsyCap, which, in turn, will result in higher self‐compassion*.

## 3 Methodology

We employed a quantitative research method to investigate relations between social support, self‐compassion and PsyCap.

### 3.1 Participants

The sample (*N* = 154) comprised 88 males (57%) and 66 females (43%). Forty‐two participants self‐identified as siblings of individuals with developmental disabilities, and 112 were siblings of individuals without developmental disabilities. Participants reported between one and 10 siblings (M = 4). Seventy (45%) were employed full‐time, 41 (27%) were employed part‐time, 19 (12%) were self‐employed and 24 (16%) were unemployed. The number of siblings ranged from 1 to 10, with an average of 4. The age of participants varied from 18 to 71 years, with a mean of 33; 101 participants (66%) were single, 48 (31%) were married, three (2%) were divorced and two (1%) were widowed. Among the siblings of individuals with developmental disabilities, 25 (16%) reported their siblings with a developmental disability were younger, and 17 (11%) reported an older siblings. Table 1 gives demographic information.

**TABLE 1:** Demographic information.

Variables	Subscale	Sibling with SN (*n* = 42)	Sibling without SN (*n* = 112)
Gender	Male	8 (19%)	80 (71%)
	Female	34 (81%)	32 (29%)
Work status	Full‐time	18 (43%)	52 (46%)
	Part‐time	12 (29%)	29 (26%)
	Independent	5 (12%)	14 (13%)
	Unemployed	7 (16%)	17 (15%)
Sibling number	Sibling range	1–9 (M = 4)	1–10 (M = 4)
Age	Age range	19–71 (M = 34)	18–71 (M = 33)
Marital status	Single	27 (64%)	74 (66%)
	Married	13 (32%)	35 (31%)
	Divorced	1 (2%)	2 (2%)
	Widowed	1 (2%)	1 (1%)
Sibling diagnosis	ASD	19 (44%)	—
	Intellectual disability	23 (56%)	—
Was your sibling with special needs younger or older than you?	Younger	25 (60%)	—
	Older	17 (40%)	—

*Note:* The category ‘intellectual disability’ refers to participants who reported an intellectual disability without specifying a particular aetiology or syndrome.

Abbreviations: ASD = autism spectrum disorder; SN = special needs.

### 3.2 Participant Recruitment

Participants were recruited using a convenience sampling strategy. To be eligible for participation, individuals must be at least 18 years old and have at least one sibling. Participants classified in the developmental‐disability group self‐identified as having a sibling with a developmental disability and provided details about the sibling's diagnosis. In this study, the reported diagnoses included autism spectrum disorder (ASD) and intellectual disability. The study invitation, which included a brief description of the study and a link to an anonymous Qualtrics questionnaire, was disseminated through social media platforms (primarily Facebook and WhatsApp) and personal networks using a snowball sampling approach. Individuals who met the inclusion criteria were invited to participate voluntarily and were encouraged to share the survey with other eligible participants. No financial or other incentives were offered for participation.

### 3.3 Instruments

#### 3.3.1 Demographic Questionnaire

Participants were asked to provide their gender, work status, number of siblings, age and marital status. The questionnaire also inquired about the presence of siblings with a developmental disability, the diagnosis and whether the siblings were younger or older.

#### 3.3.2 Psychological Capital Questionnaire (PCQ)

The PCQ is a validated tool designed to measure self‐efficacy, hope, resilience and optimism. The original PCQ‐24, developed by Luthans et al. (2007), has been widely applied in organizational psychology. To streamline the assessment process, Avey et al. (2011) introduced a 12‐item version (PCQ‐12), which maintains strong psychometric properties. The PCQ‐12 consists of 12 items, each rated on a 5‐point Likert scale from 1 (*strongly disagree*) to 5 (*strongly agree*). The PCQ‐12 has demonstrated robust internal consistency, with Cronbach's alpha coefficients ranging from 0.70 to 0.85 (e.g., Avey et al. 2011; Dawkins et al. 2013). Factor analyses consistently support the four‐factor structure, confirming the distinctiveness of each psychological capacity (Dawkins et al. 2013; Lorenz et al. 2016). Validity assessments indicate the PCQ‐12 possesses good convergent validity (Avey et al. 2011; Rego et al. 2012) and good discriminant validity. Criterion‐related validity is evidenced by positive associations with desirable outcomes, such as job satisfaction and performance, and negative associations with job stress and turnover intentions (Avey et al. 2010; Luthans et al. 2008). These findings underscore the PCQ‐12's reliability and validity as a concise measure of PsyCap in diverse contexts. Note that although the questionnaire comprises several factors, it is commonly treated and analysed as a single measure, wherein the whole is greater than the sum of its parts (Avey et al. 2011; Luthans and Youssef‐Morgan 2017). Cronbach's alpha in our study was 0.88.

#### 3.3.3 Social Support Questionnaire (SSQ)

The SSQ, developed by Zimet et al. (1988), is a well‐established multidimensional tool designed to assess perceived social support from family, friends and significant others. It consists of 12 items, each rated on a 7‐point Likert scale from 1 (*a situation not applicable to the respondent*) to 7 (*a situation highly applicable*). The responses are averaged to provide an overall score, with higher scores indicating greater perceived social support. The SSQ has been widely used in diverse research contexts because of its robust psychometric properties. It has high internal consistency, with Cronbach's alpha typically exceeding 0.85 for the total scale and its subscales (Zimet et al. 1988). Test–retest reliability has shown strong stability over time. The SSQ has also demonstrated good convergent validity. It correlates positively with other measures of social support and related constructs such as life satisfaction and mental health, indicating it effectively captures the construct of social support (Dahlem et al. 1991; Zimet et al. 1988). Its discriminant validity is supported by low correlations with unrelated constructs, ensuring it measures social support distinctly from other psychological constructs. Criterion‐related validity has been established through its associations with various outcomes. High perceived social support, as measured by the SSQ, has been linked to better mental health outcomes, including lower levels of anxiety, depression and stress, as well as higher levels of well‐being and life satisfaction (Canty‐Mitchell and Zimet 2000; Zimet et al. 1988). Cronbach's alpha in our study was 0.91.

#### 3.3.4 Self‐Compassion Scale (SCS)

The SCS, developed by Neff (2003b), is a validated instrument designed to measure self‐compassion, encompassing six dimensions: self‐kindness, self‐judgement, common humanity, isolation, mindfulness and overidentification. The scale consists of 26 items, with subscale scores computed by calculating the mean of subscale item responses. For the overall self‐compassion score, the negative subscale items (self‐judgement, isolation and overidentification) are reverse‐scored before calculating the total score. The SCS has demonstrated high internal consistency, with Cronbach's alphas typically ranging from 0.75 to 0.94 for the total score and the subscales (Neff 2003a; Neff and E. Pommier 2013). Test–retest reliability has also been shown to be robust, indicating the scale reliably measures self‐compassion over time (Neff 2003a). The SCS has strong construct validity, evidenced by positive correlations with measures of psychological well‐being, such as life satisfaction, happiness and emotional intelligence (Neff 2003a; Neff and Vonk 2009). Convergent validity is supported by significant positive correlations with other measures of self‐compassion and related constructs like self‐esteem and social connectedness (Neff 2003a; Neff and E. Pommier 2013). Discriminant validity is established through low correlations with unrelated constructs, ensuring the SCS measures self‐compassion distinctly from other psychological traits. Factor analyses consistently support the six‐factor structure, confirming the distinctiveness of each dimension (Neff 2003a; Neff and E. Pommier 2013). The SCS has demonstrated criterion‐related validity, with high self‐compassion scores associated with lower levels of anxiety, depression and stress, and higher levels of psychological well‐being and adaptive coping strategies (Neff 2003a; Neff and Germer 2013). Cronbach's alpha in our study was 0.91.

### 3.4 Research Procedure Overview and Data Analysis

Participants who accessed the survey link were first presented with an information page describing the purpose of the study, the voluntary nature of participation and assurances of anonymity and confidentiality, thus aligning with ethical research standards. Participants were informed they could withdraw at any stage of the questionnaire completion process. After providing electronic informed consent, participants completed the online questionnaire using the Qualtrics platform. Completing the questionnaire took approximately 10 min.

The research design was cross‐sectional, and data were collected over 5 months. The cross‐sectional design provided a snapshot of the variables at a single point in time.

Data analysis was conducted using SPSS software, 28th edition. We used *t*‐tests to explore differences between siblings of individuals with and without developmental disabilities in mean levels of PsyCap, self‐compassion and social support, as well as the subscales of these variables. We calculated Pearson correlations to assess the strength and direction of relations between PsyCap, self‐compassion and social support. These analyses helped us to identify potential patterns and interactions. Finally, we used the Hayes' PROCESS macro for SPSS (Version 4.2; Hayes 2022), Model 4, procedure to examine the mediating role of PsyCap in relations between self‐compassion and social support.

## 4 Results

### 4.1 Correlations Between PsyCap, Support and Self‐Compassion

Our first research hypothesis was that we would find positive correlations between PsyCap, social support and self‐compassion at the sample level and for each group separately. We used Pearson correlations to test this hypothesis. We found statistically significant correlations between PsyCap, support and self‐compassion for the entire sample and for siblings of individuals with developmental disabilities: in both groups, the higher the PsyCap, the higher the support and the higher the self‐compassion. However, for siblings of individuals with developmental disabilities, we only found statistically significant correlations between PsyCap and self‐compassion: the higher the PsyCap, the higher the self‐compassion. No other significant associations were found (Table 2).

**TABLE 2:** Correlations between PsyCap, support and self‐compassion.

Variables	PsyCap	Support	Self‐compassion
Entire sample			
*PsyCap*			
Support	0.415***		
Self‐compassion	0.483***	0.294**	
Sibling with developmental disabilities			
*PsyCap*			
Support	0.263		
Self‐compassion	0.533***	0.292	
Sibling without developmental disabilities			
*PsyCap*			
Support	0.473***		
Self‐compassion	0.467***	0.302*	

* *p* < 0.05.

** *p* < 0.01.

*** *p* < 0.001.

### 4.2 Differences Between Siblings of Individuals With and Without Developmental Disabilities

We hypothesized that there would be differences between the groups in PsyCap, social support and self‐compassion, whereby siblings of individuals without developmental disabilities would have lower levels of PsyCap, self‐compassion and social support. We conducted *t*‐tests for two independent samples. No statistically significant differences emerged (Table 3).

**TABLE 3:** Differences in variables based on having a sibling with developmental disabilities.

Variables	Sibling with developmental disabilities	*n*	M	SD	*t*
PsyCap	Yes	42	3.89	0.55	*t*(110) = 1.43, *p* = 0.155
	No	70	3.73	0.61	
Support	Yes	42	6.04	0.87	*t*(110) = 0.90, *p* = 0.370
	No	70	5.86	1.08	
Self‐compassion	Yes	42	3.11	0.63	*t*(110) = −0.17, *p* = 0.862
	No	70	3.14	0.61	

### 4.3 PsyCap as Mediator of Support and Self‐Compassion

We hypothesized that relations between social support and self‐compassion would be mediated by PsyCap, whereby greater social support would lead to higher PsyCap, which, in turn, would result in higher self‐compassion. To examine the proposed mediation model, we conducted regression‐based mediation analyses using Hayes' PROCESS macro for SPSS (Version 4.2; Hayes 2022), Model 4. Indirect effects were estimated using 5000 bias‐corrected bootstrap samples, and 95% bias‐corrected confidence intervals (CIs) were calculated. An indirect effect was considered statistically significant when the CI did not include zero (Table 4; Figure 1). The direct effect of support on self‐compassion was statistically insignificant. Tests of the indirect effects of support on self‐compassion via PsyCap were significant (B = 0.111, SE = 0.039, CI; 0.045–0.196). Higher support predicted higher PsyCap, which, in turn, predicted higher self‐compassion.

**TABLE 4:** Mediation between support and self‐compassion via PsyCap.

Predicted variable	Predicting variable	B	SE	LLCI	ULCI
PsyCap (*R* ^2^ = 17.2%)	Support	0.243	0.051	0.143	0.344
Self‐compassion (*R* ^2^ = 24.4%)	Support	0.069	0.056	−0.042	0.181
	PsyCap	0.456	0.096	0.266	0.645

**FIGURE 1:**
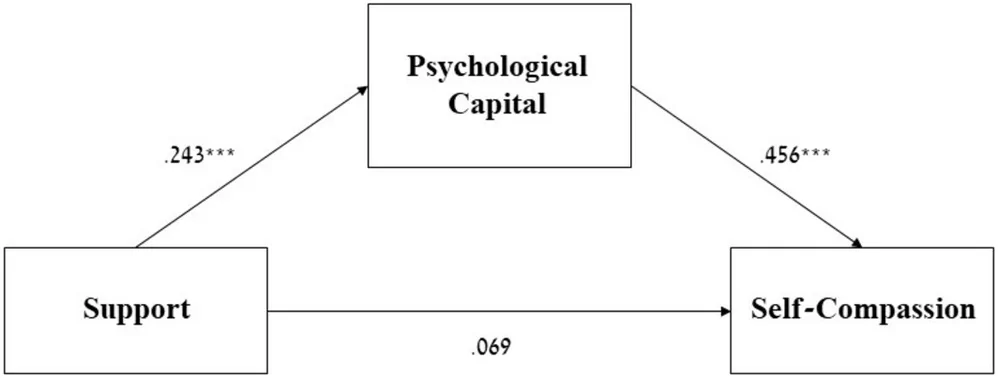
Mediation between support and self‐compassion via PsyCap.

## 5 Discussion

Families of individuals with developmental disabilities have challenges that significantly impact all family members, particularly siblings. Despite the importance of understanding these dynamics, research on the experiences and psychological well‐being of siblings of individuals with developmental disabilities remains limited. We began to fill this gap by comparatively exploring relations between PsyCap, social support and self‐compassion among siblings of individuals with and without developmental disabilities. We hypothesized that we would find positive correlations between PsyCap, social support and self‐compassion, both at the sample level and within each group (siblings of individuals with and without developmental disabilities). We further hypothesized that we would find differences between groups in levels of PsyCap, social support and self‐compassion, whereby siblings of individuals with developmental disabilities would have higher levels. Finally, we hypothesized that relations between social support and self‐compassion would be mediated by PsyCap in both groups, with greater social support leading to higher PsyCap, resulting in higher self‐compassion.

Findings corroborated the first hypothesis. We found statistically significant correlations between PsyCap, social support and self‐compassion across the entire sample. Participants with higher PsyCap reported higher levels of social support and self‐compassion. This pattern held for the whole sample and for siblings of individuals without developmental disabilities. For siblings of individuals with developmental disabilities, we only found statistically significant correlations between PsyCap and self‐compassion, not social support. This distinct pattern suggests the development of internal psychological resources is crucial for fostering self‐compassion in this group, possibly because of their unique stressors and responsibilities.

We hypothesized that siblings of individuals with and without developmental disabilities would have different levels of PsyCap, social support and self‐compassion, but we did not find statistically significant differences. Being the sibling of an individual with developmental disabilities may not necessarily impact levels of PsyCap, social support and self‐compassion in the ways hypothesized (Garrido et al. 2020). These siblings may develop comparable levels through other means or compensatory mechanisms. The finding could also reflect the variability in individual experiences and the quality of social support received.

Hypothesis 3 was corroborated. The correlations we observed between PsyCap, social support and self‐compassion for all participants align with prior research (Lowery and Cassidy 2022; Poots and Cassidy 2020). The mediation effect can be understood through several mechanisms. By providing emotional, informational and instrumental assistance, social support helps individuals build and maintain psychological resources. These resources, in turn, enhance PsyCap by boosting self‐efficacy, optimism, hope and resilience. PsyCap, as a positive psychological state, enables individuals to approach challenges with greater confidence and positivity. This positive state of mind fosters self‐compassion, allowing individuals to treat themselves with kindness during difficult times.

Our findings for PsyCap are consistent with the work of Luthans and Youssef‐Morgan (2017), who describe PsyCap as a state. This state is not innate but develops through exposure to life experiences, both positive and negative. The development of PsyCap is crucial for fostering resilience and a proactive mindset, particularly for siblings of individuals with developmental disabilities, who may face unique stressors and responsibilities. Our findings align with Neff and Davidson (2016), who explored self‐compassion as a critical factor in psychological well‐being, influencing self‐esteem, motivation, health, body image and interpersonal relationships. For siblings of individuals with developmental disabilities, self‐compassion serves as a protective factor, mitigating the adverse effects of stress and enhancing psychological resilience.

### 5.1 Implications for Interventions

The findings have important implications for interventions supporting siblings of individuals with developmental disabilities. The observed associations among perceived social support, PsyCap and self‐compassion suggest that interventions should strengthen both external support networks and internal psychological resources. As PsyCap is considered a developable psychological resource, structured interventions targeting its components may directly enhance the psychological well‐being and self‐compassion of siblings (Luthans et al. 2015).

Sibling‐specific interventions already include psychoeducational programmes, peer support groups, family‐centred services and established programmes such as Sibshops, providing crucial opportunities for peer support, emotional expression and adaptive coping (Hartling et al. 2010; Meyer and Vadasy 2008; Meltzer 2021; Roberts et al. 2015). However, we addressed a critical gap in the literature: Research has established the general benefits of these interventions, but relatively little work considers the underlying psychological mechanisms through which such support contributes to the well‐being of siblings.

By demonstrating PsyCap fully mediates relations between social support and self‐compassion, this study is among the first to isolate PsyCap as the core internal mechanism necessary for translating external support into emotional well‐being among siblings of individuals with developmental disabilities. Consequently, the findings add to existing support programmes by providing a clear, empirical roadmap: Rather than focusing solely on facilitating peer interactions, programmes can integrate explicit PsyCap‐building strategies, such as resilience training and positive psychology interventions within educational and healthcare settings, to systematically foster internal positive psychological functioning.

The implementation of these interventions must be sensitive to developmental stage and age. There may be distinct differences in how PsyCap and social support are fostered across the lifespan, as younger siblings and adult siblings encounter different emotional, social and caregiving challenges (Meltzer 2021). Because caregiving demands and the use of social networks shift as siblings mature, developmentally tailored interventions are essential. Age‐sensitive strategies would ensure the methods used to cultivate PsyCap remain responsive to siblings' evolving needs and unique age‐related stressors across the lifespan.

### 5.2 Strengths, Limitations and Future Research

This study has several notable strengths. To the best of our knowledge, it is one of the first to examine relations between perceived social support, PsyCap and self‐compassion among siblings of individuals with developmental disabilities. By integrating these positive psychological constructs within a single mediation model and including a comparison group of siblings of individuals without developmental disabilities, the study contributes to a more comprehensive understanding of the protective factors promoting sibling well‐being.

Nevertheless, several limitations should be acknowledged. First, the cross‐sectional design precludes causal inferences regarding the observed relations. Second, the use of self‐report questionnaires may have introduced response and social desirability biases. Third, participants were recruited through convenience and snowball sampling using social media, possibly limiting the generalizability of the findings.

Future longitudinal research should explore how these relations evolve over time and examine the long‐term impact of strengthened PsyCap. Qualitative studies may offer a deeper understanding of the lived experiences of these siblings, including how they perceive and utilize social support. Investigating potential moderating factors, such as the nature of the individual's developmental disability, the availability and quality of social support and personal differences in coping strategies, would enhance our understanding of these dynamics and contribute to developing more effective, tailored interventions.

## 6 Conclusion

This study highlights the critical role of PsyCap in fostering self‐compassion among siblings of individuals with developmental disabilities while noting the less prominent role of social support in this relationship. We underscore the importance of developing internal psychological resources in this demographic and call for targeted interventions to enhance PsyCap. However, further research is needed to deepen our understanding of these dynamics and to inform the creation of more effective support strategies.

## Author Contributions

**Batel Hazan‐Liran:** conceptualization, investigation, writing – original draft, methodology, writing – review and editing, formal analysis, data curation. **Ofra Walter:** investigation, writing – original draft, writing – review and editing, methodology.

## Funding

The authors have nothing to report.

## Ethics Statement

All procedures followed were in accordance with the ethical standards of standards IRB of the Tel Hai institutional and national committee and with the Helsinki Declaration of 1975, as revised in 2000. Informed consent was obtained from all patients included in the study.

## Conflicts of Interest

The authors declare no conflicts of interest.

## Data Availability

The data that support the findings of this study are available from the corresponding author upon reasonable request.
